# Management of Dehydration in Patients Suffering Swallowing Difficulties

**DOI:** 10.3390/jcm8111923

**Published:** 2019-11-08

**Authors:** Emilie Reber, Filomena Gomes, Ilka A. Dähn, Maria F. Vasiloglou, Zeno Stanga

**Affiliations:** 1 Department for Diabetes, Endocrinology, Nutritional Medicine and Metabolism, Bern University Hospital, and University of Bern, Freiburgstrasse 15, 3010 Bern, Switzerland; zeno.stanga@insel.ch; 2 The New York Academy of Sciences, 250 Greenwich Sweet, 40th floor, New York, NY 10007, USA; filomenisabel@hotmail.com; 3 Cereneo Schweiz AG, Center for Neurology and Rehabilitation, Seestrasse 18, 6354 Vitznau, Switzerland; ilka.daehn@cereneo.ch; 4 AI in Health and Nutrition Laboratory, ARTORG Center for Biomedical Engineering Research, University of Bern, Murtenstrasse 50, 3008 Bern, Switzerland; maria.vasiloglou@artorg.unibe.ch

**Keywords:** dehydration, dysphagia, fluid intake, water

## Abstract

Swallowing difficulties, also called dysphagia, can have various causes and may occur at many points in the swallowing process. The treatment and rehabilitation of dysphagia represent a major interdisciplinary and multiprofessional challenge. In dysphagic patients, dehydration is frequent and often accelerated as a result of limited fluid intake. This condition results from loss of water from the intracellular space, disturbing the normal levels of electrolytes and fluid interfering with metabolic processes and body functions. Dehydration is associated with increased morbidity and mortality rates. Dysphagic patients at risk of dehydration thus require close monitoring of their hydration state, and existing imbalances should be addressed quickly. This review gives an overview on dehydration, as well as its pathophysiology, risk factors, and clinical signs/symptoms in general. Available management strategies of dehydration are presented for oral, enteral, and parenteral fluid replacement.

## 1. Introduction

Dysphagia is a dysfunction of the digestive system, consisting of a difficulty in swallowing. It affects the proper transit of the bolus in the upper digestive tract, preventing a safe oral feeding process. The main complications of dysphagia are aspiration (i.e., the passage of solid and liquid food into the respiratory tract, which can be “silent” in the absence of the protective cough reflex), aspiration pneumonia (pneumonia caused by food in the lungs), malnutrition, and dehydration. Because symptoms are very unspecific, dysphagia is frequently undetected, and either untreated or undertreated. Nevertheless, prevalence of dysphagia is high, especially among older patients with neurological disorders due to multifactorial changes of swallowing physiology, affecting at least 50% of the acute stroke population and 60% of those who suffer a severe traumatic brain injury [1,2]. In a recent systematic review with six high quality studies, the average prevalence of dysphagia in the community dwelling elderly population was 15% [3]. The same authors defined the risk factors for dysphagia as a history of clinical disease, physical frailty, and reduced ability to accomplish activities of daily living [3]. Dysphagia and the subsequent reduction of the coordination of pharyngeal muscles increase the risk of dehydration, malnutrition, pneumonia, and mortality [2]. Consequently, dysphagia may lead to dehydration—a shortage of body water due to either insufficient drinking or excess losses, or a combination of both [4]. Water and electrolyte balance is crucial for body homeostasis and is one of the most protected mechanisms in the body. Organisms can survive months without eating, but not many days without drinking, as fluid and electrolytes play major regulatory roles in many mechanisms, for example, transport systems, signal transduction, and body temperature [5].

This review highlights and discusses dehydration, as well as its pathophysiology, risk factors, and clinical signs/symptoms, focusing on dysphagic patients in particular. Possible management strategies of dehydration are presented for oral, enteral and parenteral fluid replacement.

## 2. Dysphagia

Swallowing is an essential and highly complex process of movements. This physiological process requires an exact coordination of nerval and muscular structures as well as an intact sensitivity of the mouth and throat area. The deglutition can be divided into five phases (preoral, two oral phases, pharyngeal, and oesophageal), whereby the phases run smoothly into each other and work together as muscle chains. Normally, the act of swallowing proceeds unnoticed and highly automated [6,7]. The muscle strength and the temporal orientation of the movement sequence are regulated in relation to the food/drink in the mouth (consistency and quantity). The system can be severely disturbed by diseases such as strokes, degenerative diseases, traumas, or tumours. A swallowing disorder (dysphagia) can occur in different forms. A healthy person swallows about 1,000 to 2,000 times a day [8]. The adaptation of food/drink, for example, balanced soft food or thickened liquids, may be needed to enable the patient to eat orally without an additional risk of aspiration.

Motor and cognitive performance decreases with increasing age [9,10]. Changes in the age-related swallowing disorder (presbyphagia) are the reduction of the sense of taste and smell, a reduced chewing function due to lack of oral hygiene/prosthesis, and the loss of strength to swallow food and liquids effectively and safely [10]. More than 40% of muscle mass is lost in old age [11]. Cancer patients are also severely impaired in their food intake owing to severe surgery, subsequent chemotherapy, and/or radiation. All these factors make the patients highly dependent [9,12], which leads to further health problems such as dehydration, malnutrition, anorexia, sarcopenia, and pneumonia. Older people and patients with dementia are more susceptible to stroke, pneumonia, and dehydration, which are among the most common causes of death in this population [9].

Dysphagia is associated with malnutrition, dehydration, pneumonia, reduced functional outcome, and mortality [13]. Therefore, similarly to nutritional management, populations at risk should regularly be screened for dysphagia and, when needed, further assessed by a clinical specialist (e.g., a speech-language pathologist). Patients suspected to have dysphagia after a screening test undergo assessment to determine the degree of severity of dysphagia and further treatment. Individual screening tests are performed by speech-language pathologists or trained nursing staff. They serve to identify symptoms and differentiate dysphagic patients from non-dysphagic patients. This procedure is non-invasive and can be performed quickly (in 10–15 min) while the patient is sitting in an upright position. There are several criteria that indicate that the screening test should be ceased in order to minimize the risk of aspiration (i.e., the involuntary passage of food or liquid through the vocal folds, to the lungs). Nonetheless, this test shows a sensitivity and specificity between 50% and 80% and fails to detect a significant number of aspirations [14,15].

The clinical swallowing examination is performed by speech-language pathologists. The patient sits upright at 90°. The inspection of the facial-oral structures, the oral skills, and sensitivity, as well as the oral motor arbitrary movements; the oral reflexes in the pharynx area (gag reflex, palata reflex, and so on); and the existing protective mechanisms, such as coughing and deliberate coughing, are included [16]. According to Daniels et al., liquid is gradually swallowed, thus single sips ≤20 mL of water up to 100 ml are examined (timed test) [17]. Soft and solid consistencies are also examined. Pathomechanisms like leaking anterior, nasal penetration, signs of aspiration, and residuals in the oral cavity are observed and described. Finally, an inspection of the oral cavity and a longer phonation sample are performed to clarify a sizzling voice and possibly the suspicion of penetration with aspiration [16]. In addition, instrumental examinations such as, for example, fiber optic endoscopic evaluation of swallowing (FEES; a transnasal procedure) and the video fluoroscopic swallowing study (a radiographic procedure), can be carried out.

FEES is a reportable procedure that may be completed in an outpatient clinic setting or at the bedside by passing an endoscope transnasally. It requires minimal positioning of the patient, and is thus commonly used [18]. In contrast, the video-fluoroscopy swallowing study, the gold standard for diagnosis of dysphagia, is a radiographic procedure that provides a direct, dynamic view of oral, pharyngeal, and upper oesophageal function. It, however, requires the use of a radiology suite (which can be very costly) and includes radiation exposure of the patient, who must be able to follow verbal commands (i.e., requires adequate cognitive functioning) [18]. All results will be brought together and the individual therapy plan will be defined. Regular repetitive examinations are needed to adapt swallowing treatment and the nutrition and hydration care plan.

Dysphagia therapy may be split into two categories: compensatory and rehabilitative therapies. Compensative strategies aim to maintain patient’s safety when eating, while rehabilitative strategies aim to speed up the recovery process (e.g., swallowing training). Patients may be treated first with compensatory therapy and later with rehabilitation treatments. Functional oriented swallowing therapy, defined and applied by a trained speech-language pathologist, aims to create muscular conditions for largely normal swallowing and, therefore, to recover the physiological swallowing function, maintaining or improving quality of life by reducing morbidity and mortality associated with chest infections (pneumonia) and reduced nutritional status. There are also several supportive technical stimulation treatment methods available such as thermal tactile stimulation, transcranial magnetic stimulation, or transcranial direct current stimulation [19].

On the basis of the severity of dysphagia complaints and the level of alertness, the adequate food support with modified texture diets and thickened fluids will be proposed. The change of food consistency is mandatory in dysphagia, in order to reduce the need of oral manipulation, to make the swallowing process slower and safer, and to increase food and fluid intake [20]. Nevertheless, such diets lean towards less acceptance by patients, less nutritionally dense meals, less food choices, and less appealing meals than normal, leading to insufficient nutritional and fluid intake [21,22]. That is why it makes sense to make use of food fortification as well as supplemental enteral feed and fluids in these patients. Thickening agents, which are used to thicken a variety of fluids (e.g., water, tea, coffee, fruit juice), are supposed to induce a “coating feeling” in the mouth, suppressing flavour without reducing the sensation of thirst [23]. Hence, swallowing safety may be improved, but fluid intake does not substantially improve [1]. Liquid thickening is recommended in patients with dysphagia aspirating on liquids, as a way to slow the flow of the swallowed liquids, which should allow more time for airway closure and eventually reduce the risk of aspiration [24,25]. However, thickening fluids significantly decreases of the acceptance of the beverages, and thus is a practice that needs to be closely monitored. Whelan et al. showed in their study of 24 acute stroke patients with dysphagia that the intake of thickened fluids per day amounted to only 30% of the recommended 1500 mL/day [22]. The other 70% of the hydration needs had to be met through supplementary (parenteral and enteral) fluids. The group of patients who received pre-thickened fluids consumed almost double the amount of fluids than the group of patients receiving powder-thickened fluids, suggesting that the commercially available pre-thickened ready-to-drink beverages have better acceptability and can be used as a strategy to increase fluid intake among dysphagic patients (although the costs may he higher) [22].

Different food consistencies have been defined and numerous guidelines exist in many countries describing the various types of texture-modified diets and thickened fluids. These recommendations, aiming to improve patient’s safety and nutritional status while avoiding aspiration pneumonia, are, however, based on best practice and are not evidence-based [26,27]. Modification of food texture and liquid thickness is a mainstay of dysphagia management. As there are only a few high-quality studies, the evidence is weak [28]. A couple of years ago, the “International Dysphagia Diet Standardisation Initiative” (IDDSI) published new global standardized definitions describing texture-modified foods and thickened liquids [29]. This terminology allows the use of a common language on multiprofessional teams (therapists, nurses, chiefs, patients, and relatives), which will support scientists working in this field, thus generating comparable research data worldwide. When applying the IDDSI definitions in each clinical setting, the food and drinks should always be tested under serving conditions. Drinks and liquidized foods are evaluated by a gravity flow test with a 10 ml syringe. This classifies beverages based on their flow rate, representing the process of drinking through a straw [29].

It is important to note that thickened liquids and adapted food consistencies are only a start to increase the quality of life of the dysphagic patients, as they work as a motivation tool and prepare them to eventually swallow food and fluid with normal consistency. Given the disadvantages of the use of thickened fluids and adapted food consistencies (which may lead to insufficient nutritional and fluid intake), it is important to control the progression of the dysphagia in order to adapt the food/drink consistency and protect the patient from being restricted to a certain consistency for too long [30].

## 3. Dehydration

Adequate water supply is indispensable to maintain cellular homeostasis and several physiological functions. Historically, total body water loss is differentiated into two types: dehydration and volume depletion [31]. Dehydration occurs when the body water losses, mostly from the intracellular volume (ICV), are higher than the intake. Low-intake dehydration is a shortage of pure water, leading to loss of both intracellular and extracellular fluid, and to raised osmolality in both compartments. Volume depletion is the result of excess losses of fluid and salts (especially sodium and sometimes other components), primarily related to a loss of extracellular volume (ECV), and clinically affecting the interstitial compartment; the fluid is lost primarily, not intracellular fluid, and serum osmolality will be normal or low. From the physiological view point, it makes sense that many physicians tend to use the term of dehydration for any loss of total body water in daily clinical practice [32].

There are numerous definitions for dehydration hampering the diagnosis. The best diagnostic approach for this complex condition thus includes history, clinical observations, laboratory tests, and physical assessment [33]. From a clinical point of view, dehydration may be defined as the rapid decrease of >3% of body weight [34]. Pathophysiologically, however, dehydration is a loss of water, resulting in a relative deficit of body water referring to sodium [35]. Consequently, increased sodium values cause the plasma osmolality to rise, reducing the ICV. This is often referred to as hypovolemic hypernatremia or hypertonic dehydration. Even if commonly used, “isotonic dehydration” and “hypotonic dehydration” differ from a pathophysiologic point of view, rather characterizing a volume depletion (loss of sodium from the ECV) than dehydration [35]. This distinction is especially relevant for the therapeutic approach [33].

If the fluid intake is too low, fluid in and around body cells concentrates, raising plasma and serum osmolality [36]. This consecutively triggers protection mechanisms (e.g., thirst and increased urine concentration by the kidney). In the elderly, with kidney function being mostly low, renal parameters do not truthfully indicate dehydration [36]. The U.S. Panel on Dietary Reference Intakes for Electrolytes and Water hence considers the plasma or serum osmolality as the main factor of hydration status, thus setting the reference standard for dehydration in older adults [37]. This is based on physiological and biochemical considerations and is accepted by experts [36]. Extracellular water loss due to diarrhoea, vomiting, or renal sodium loss (volume depletion) is, however, linked to normal or decreased plasma osmolality. While severe dehydration is not exactly defined, there are indicators of a distinctive lack of water: serum osmolality ≥300 mOsm/kg, serum sodium concentration ≥150 mmol/L, or blood urea nitrogen (BUN) to creatinine ratio ≥20. In the case of hypernatremia, free water shortage may be estimated with the following formula [35]:

free water shortage (L) = 0.6 × body weight (kg) × ((plasma sodium (mmol/L)/140) − 1)

### 3.1. Adequate Intake

The recommendations for adequate fluid intake show great variation (from 1.0 L/day in the Nordic countries to 2.2 L/day in the USA in women, 1.0–3.0 L/day in men) [36]. The European Food Safety Authority recommends an adequate intake of 2.0 L/day for women and 2.5 L/day for men of all ages [38]. Because 80% of the fluids come from beverages, adequate intake is 1.6 L/day in women and 2.0 L/day in men. Fluid requirements need to be adapted individually, for example, increased with higher activity level, fever, diarrhoea, and vomiting, or decreased in the case of heart and renal failure.

### 3.2. Prevalence

As dehydration is not properly defined, it is problematic to accurately assess its prevalence. Dehydration is, however, especially common in the elderly (up to 60%), depending on the definition used [39]. Bennett et al. showed that laboratory parameters indicated dehydration in 48% of elderly people admitted to an emergency department, while proper assessment of dehydration was documented only in 26% [40]. Dehydration is one of the ten most frequent diagnoses for hospital admission in older adults [41], and has been reported to be the most common fluid and electrolyte imbalance in older adults [42]. A study showed that 6.7% of inpatients aged ≥65 years were diagnosed with dehydration, with it being the principal diagnosis in 1.4% [43]. Among those suffering from swallowing difficulties, the prevalence of dehydration ranges from 44% [44] to 75% depending on the patient population, setting, and criteria used to define dehydration [45]. Nevertheless, Thomas et al. revealed that physicians misdiagnose dehydration in at least 30% of older adult inpatients [46].

### 3.3. Impact and Risk Factors

Elderly people tend to have diminished feeling of thirst owing to the decrease of sensitivity to the antidiuretic hormone (ADH) [47]. Numerous studies showed that adequate hydration status is normally maintained in healthy older people. Nevertheless, mental and/or physical illnesses, trauma, or operation, among others, may increase the risk of dehydration [48]. A six-month study among nursing home residents found 31% to be dehydrated during that period, and thereof, two-thirds had prior episodes of dehydration [49]. Risk factors for dehydration in dysphagic nursing home residents were, among others, severe impairment of the functional and/or cognitive function, speech disorders [45], and insufficient support at mealtime [50]. Inadequate nursing staff training, multiple medication, and being of female gender have also further been shown to increase the risk of dehydration [51]. Elderly people affected by acute infections and or chronic diseases (cardiovascular disease, diabetes, cancers), especially polymorbid patients, are at high risk of dehydration [40,41,43,51]. Dysphagia was shown to be directly linked with dehydration [4]. Evidence has shown that the clinical outcome of elderly people is worse in the presence of dysphagia [36]. High serum osmolality (>300 mOsm/kg) has been linked to an increased disability and mortality risk [36].

**Figure 1:**
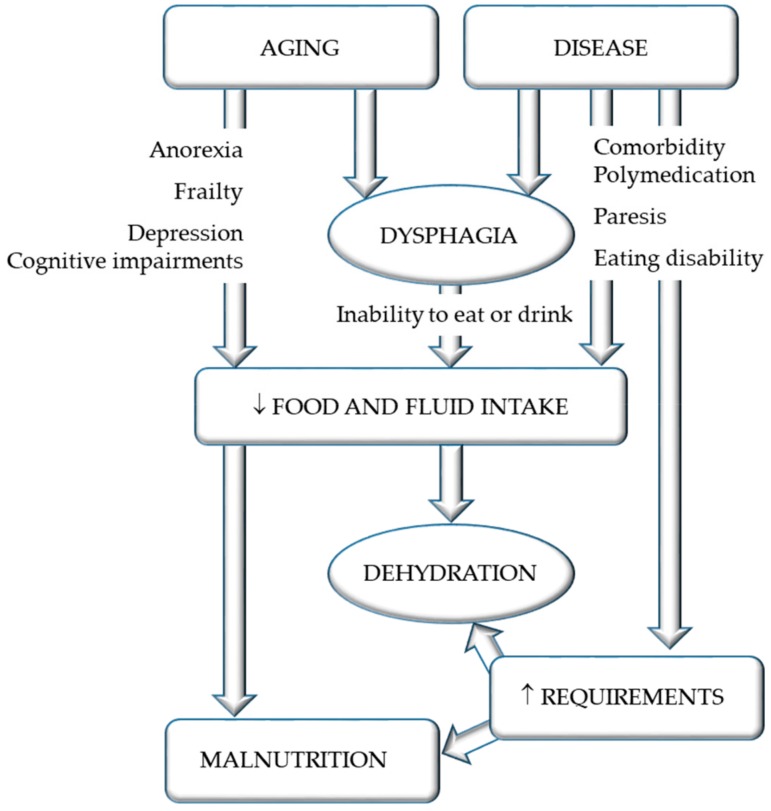
Risk factors for dehydration in dysphagia, modified after the work of [8].

There are many physiological age-related alterations increasing the risk of dehydration (Figure 1) [36]. Growing age seems to dampen the two main physiological answers to reduced fluid intake: thirst and primary urine concentration (through the kidney) [36]. Moreover, fluid reserves are decreased, as body water decreases with age. In addition, common drug therapies in older people may further aggravate fluid loss, such as diuretics and laxatives therapies [36]. However, the severity of cognitive and functional impairment appears to be more relevant than only older age [36]. A study with patients suffering from dysphagia and receiving thickened fluids assessed water supply, including that from food and drinks (thickened beverages), as well as from artificial nutrition (enteral and parenteral nutrition). This study showed that estimated fluid requirements were not met for any of the patients without the use of enteral or parenteral fluids [4]. Another surprising finding of this study is that food, not thickened beverages, provided the greatest contribution to oral fluid intake, which probably reflects the general low level of acceptance of and compliance with thickened fluids. It is very important for the medical and nursing staff to be aware not only of the risk of dehydration in patients with dysphagia, but also of its clinical signs and symptoms. In collaboration with speech-language pathologists and dietitians, an individual nutrition and hydration care plan must be placed in a timely manner, as dehydration may cause severe complications and increase medical costs, morbidity, and the mortality rate [43].

## 4. Pathophysiological Considerations

### 4.1. Body Compartments

**Figure 2:**
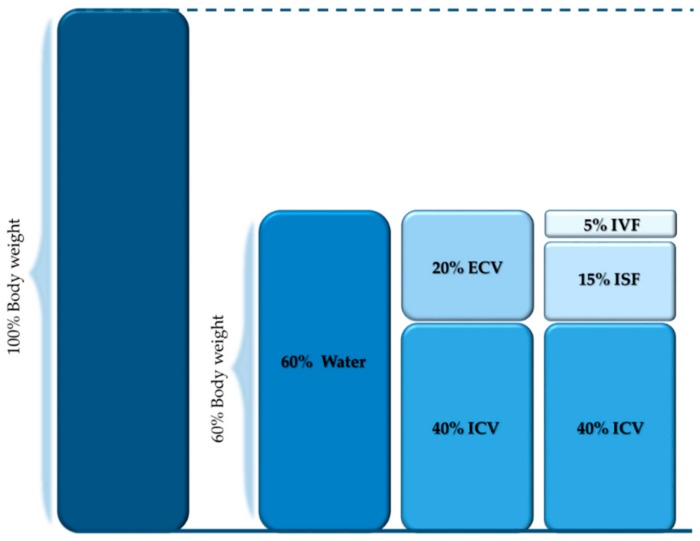
The repartition of body water within the different compartments as percentage of body weight [35]. ICV: Intracellular volume; ECV: Extracellular volume; ISF: Interstitial fluid; IVF: Intravascular fluids.

Water distribution into the different body compartments in healthy individuals is shown in Figure 2. Body water fulfils many physiological functions: it regulates body temperature and absorbs shocks, it is a solvent for chemical reactions, it serves as transport medium, and it contributes to the removal of waste products. Water accounts for about 60% of body weight. This proportion may vary according to gender and age. Body water is generally somewhat less in women than in men owing to differences in body composition. The body water is divided intracellular (two-thirds of body water) and extracellular (one-third of body water) space. The water within the extracellular space is further divided into intravascular (1/12 of body water) and extravascular (3/4 of body water) fluid by the capillary wall. The extravascular compartment may be further divided into interstitial/transcellular fluids.

### 4.2. External Fluid Balance

**Figure 3:**
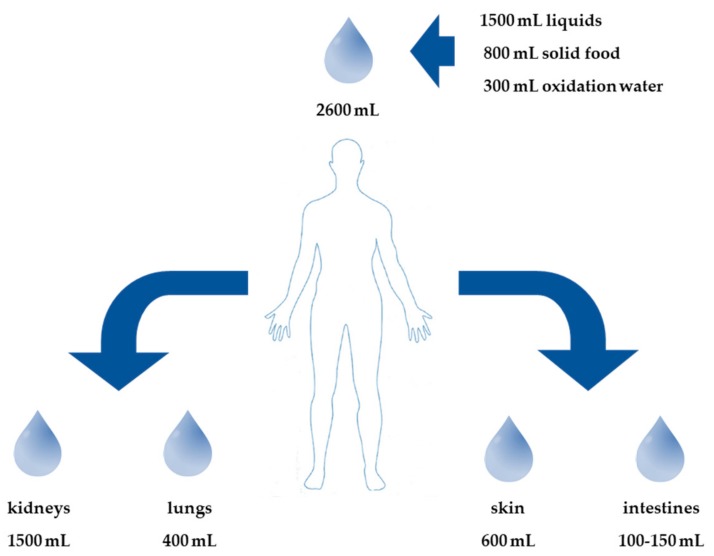
Fluid balance [35]. This figure shows the sites involved in fluid loss.

Fluid balance is a complex interplay of numerous organs (e.g., skin, respiratory tract, kidneys, and gastrointestinal tract). The typical daily fluid turnover amounts to approximately 2600 mL (30–40 mL/kg body weight). Liquids represent about 1500 mL of daily input; 800 mL comes from liquids within solid food and a further 300 mL comes from oxidation water (Figure 3). The main water output (1500 mL) passes through the kidneys. The insensible perspiration composed of water losses from respiration and transpiration accounts for a substantial loss (Figure 3), while faeces alone represent a loss of 100–150 mL per day. The regulation of the sodium balance is essential for the maintenance of normal blood volume. The regulation of the water balance, however, relies on the maintenance of osmolarity. Volume maintenance has priority at any time on osmolality. The regulation of water balance also strongly relies on the capability of the kidney to excrete urine with an osmolality different from plasma, as kidneys are very efficacious in retaining sodium, but only hardly evacuate its surplus.

### 4.3. Internal Fluid Balance

The distribution of water within the body compartments undergoes strict control. Semipermeable cell membranes separating the intracellular and extracellular space enable the passage of water and selected molecules. The quantity of solutes per kilogram of solution is referred to as osmolality and usually fluctuates around 275 to 290 mOsm/kg. It may be directly measured or calculated with the following equation:

2 × (serum sodium (mmol/L)) + BUN (mg/dL)/2.8 + plasma glucose (mg/dL)/18.

Tonicity is defined by the solutes determining the transcellular distribution of water, referred to as effective osmoles. Inactive osmole (e.g., urea) can freely pass the membrane and their rise in serum does not trigger water movement toward extracellular space. Nevertheless, inactive osmoles contribute to osmolality. Plasma osmolality is mostly determined by sodium (extracellular) and potassium (intracellular). Potassium in the extracellular fluid reaches 2% of the total body potassium. Anions such as chloride and bicarbonate and proteins grant the electroneutrality (Table 1). The first determining factor for the distribution of body water is the osmotic forces. Osmotic changes will immediately trigger the movement of water from lower to higher osmolarity, which has to be similar on both sides of the cell membrane.

## 5. Disorders of Fluid Balance

### 5.1. Isotonic and Hypotonic Dehydration 

**Figure 4:**
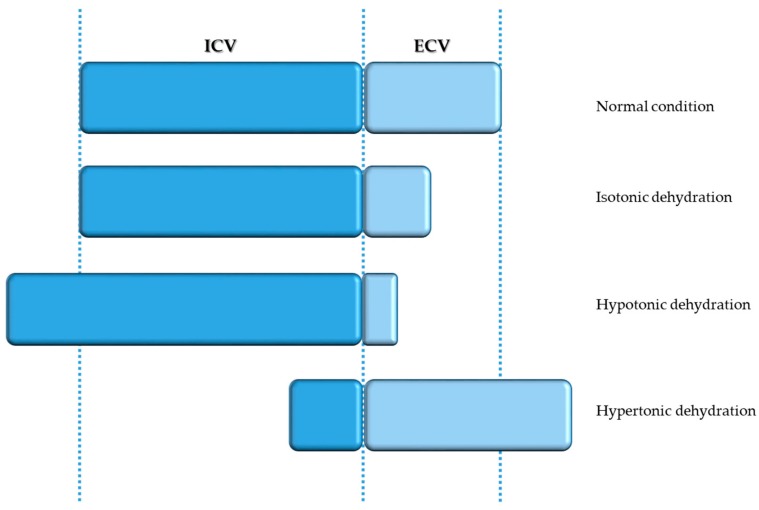
Water balance disturbances.

The term “isotonic dehydration” may be confusing to a certain degree as it in fact more refers to volume depletion than to dehydration. Isotonic dehydration occurs when the loss of water and solutes is balanced, reducing the volume of extracellular fluid, leading to reduced perfusion of the tissues, while osmolality and thus the intracellular fluid volume are kept normal (Figure 4). The reasons for this are manifold: fasting, haemorrhage, burns, gastrointestinal symptoms (vomiting, diarrhoea), drugs (sedative, diuretics), and so on. Renal perfusion is decrease owing to volume depletion and the renin–angiotensin–aldosterone system is thus activated, resulting in greater reabsorption of sodium and water. Secretion of ADH moreover causes water retention, aiming to correct volume depletion.

When sodium losses are greater than water losses, causing serum osmolality >270 mOsm/kg and sodium concentration >135 mmol/L in serum, “hypotonic dehydration” occurs. When fluids and sodium losses are partly replaced using hypotonic fluids, the extracellular space is reduced, owing to the low serum osmolality (Figure 4).

Both forms must be treated by means of isotonic fluids.

### 5.2. Hypertonic Dehydration

**Figure 5:**
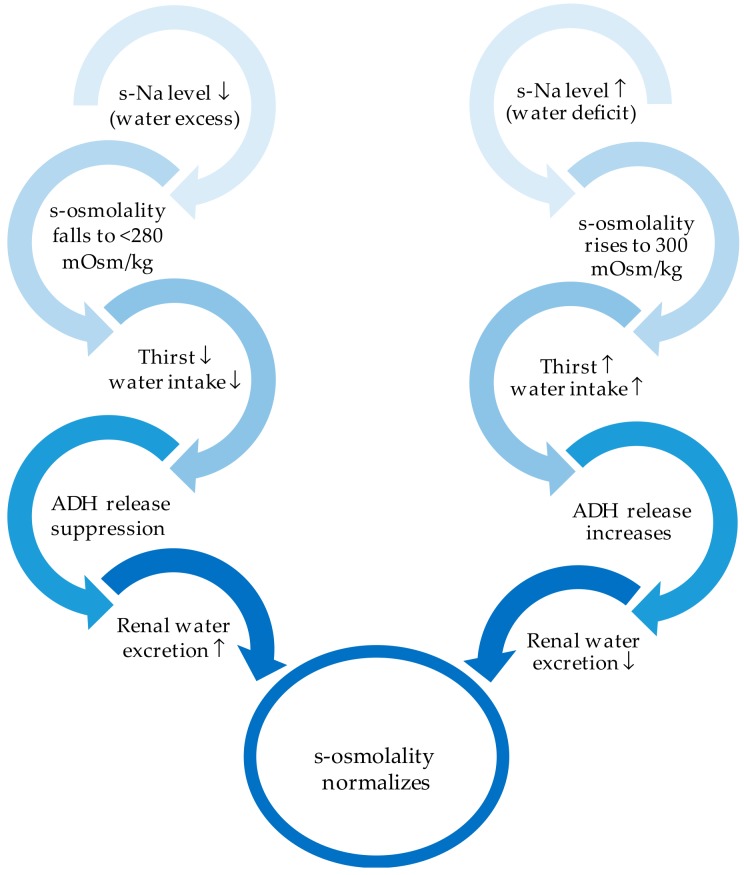
Regulation mechanisms of sodium and water. s-Na: Serum sodium; s-osmolarity: Serum osmolarity; ADH: Antidiuretic hormone.

**Figure 6:**
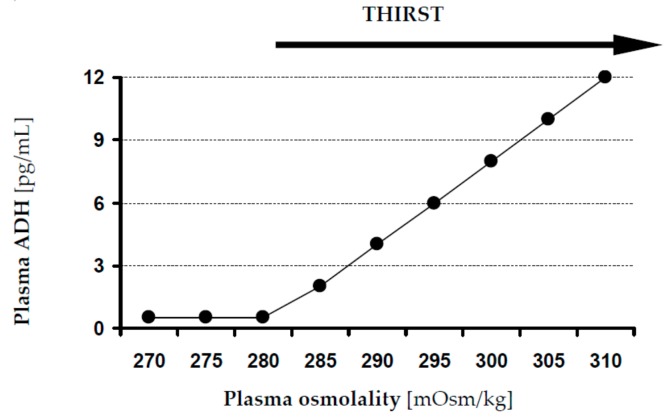
ADH release and thirst [56]. ADH: Antidiuretic hormone.

“Hypertonic dehydration” represents the pathophysiologic type of dehydration, where BUN to creatinine ratio is ≥20 and serum sodium concentrations exceed ≥150 mmol/L. Loss of water is higher than loss of sodium. The succeeding rise of sodium concentration in serum induces a rise in osmolality, which consecutively causes water to move towards the extracellular compartment, causing acute hypernatremia (Figure 4). Theses water movements also affect the brain, possibly causing a diminution in brain volume, and eventually causing rupture of veins and subsequent intracerebral or subarachnoid haemorrhage. Brain cells initially accumulate sodium and potassium, and later osmolytes (mainly myoinositol, glutamine, and glutamate), to compensate for water loss and restore volume [53,54]. As in dysphagia, hypertonic dehydration happens because of decreased water intake or as a result of either excessive (extra) renal water losses. Many factors may cause renal water losses such as renal or central diabetes insipidus, polyuric phase of acute renal failure, loop or osmotic diuretics, post obstructive disease, and so on. Extra renal water losses are the result of transcutaneous losses (e.g., sweat, fever, burns) or losses over the respiratory tract (e.g., hyperventilation). The consecutive rise in osmolality activates ADH release, and thus thirst (Figure 5). ADH secretion may be triggered by non osmolar volume-depending receptors or by supraoptic and paraventricular nuclei of the thalamus. The latest causes a reduced free water excretion. Above the threshold for ADH secretion in humans, which is 280–285 mOsm/kg, ADH secretion rises in a linear manner with rising osmolality (Figure 6). The threshold value for thirst, the major protective physiological mechanism of the body to compensate for hypernatremia, is higher than the ADH threshold [55].

Hypotonic fluids represent the best treatment option for hypertonic dehydration.

## 6. Clinical and Biochemical Diagnostic Features

The sensitivity and specificity of dehydration signs largely depend on the volume of blood loss, where the clearest signs are heart rates changes (30 beats per minute) or severe postural dizziness leading to a lack of ability to stand [57]. Medication (e.g., beta-blockers) and older age may further influence sensitivity and specificity of these signs. Signs consecutive to fluid and electrolyte losses (e.g., due to vomiting or diarrhoea) are less clear.

Clinical signs taken alone are not very useful. A combination of at least four signs described hereafter indicates moderate to severe volume depletion. Typical clinical signs of dehydration with high scientific value are listed in Table 2. Absence of tears, transpiration, and/or thirst are among others symptoms of dehydration [33,58,59]. Clinical features with poor sensitivity and specificity are as follows: dry mucous membrane, low skin turgor, long nail bed refill time, altered respiratory pattern, changes in heart rate, dry or furrowed tongue, dry axilla, absence of tears, sunken eyes, high palpated ocular pressure, and weight loss [33,57]. The highest diagnostic utility was given by systolic blood pressure <100 mmHg in a clinical trial [58]. Owing to the loss of subcutaneous tissue with aging skin, turgor is not reliable in the elderly population. If present, dry axilla is fairly used with an appositive likelihood ratio of 2.8 (sensitivity 50%, specificity 82%) [60]. Symptoms that have a sensitivity over 80% are furrows on the tongue, dry mucous membranes, speech incoherence, extremity weakness, and orthostasis. Sunken eyes show a specificity greater than 80% and a sensitivity of 62% [32]. Central nervous system symptoms like confusion, speech difficulty, or weakness in the extremities are manifest when dehydration results in a 1% loss of body weight and are more notable at a 5% loss [32,58,61]. The development of neurologic effects is more likely to occur in acute hypernatremia developing within 48 hours than in chronic cases. Severe dehydration may cause hypotension, including the related risk of falls, reduced cardiac output, as well as consecutive reduced blood flow through organs and tissues. It may also escalate to hypovolemic shock. The risk for chronic renal disease, morbidity, and mortality are increased in the case of chronic dehydration (mild to severe) if not treated [62,63,64].

Although the patient’s history and physical examination may indicate the appearance of dehydration (symptoms and signs), it is obvious that medical staff should not only rely on these clinical data, but also have to integrate biochemical parameters to enable the diagnosis (Table 2) [33,58,59]. Plasma osmolarity ≥300 mOsm/kg, plasma sodium, urine specific gravity, tear, and saliva osmolality have all been shown to be suitable to diagnose dehydration [58,65,66]. For an optimal diagnostic strategy, you need at least calculate or measure BUN, glucose, creatinine, sodium, bicarbonate, and osmolality [32].

## 7. Management of Dehydration

### 7.1. General Recommendations

The identification of acute situations potentially leading to dehydration, as well as the monitoring of vital parameters, clinical signs and symptoms, and laboratory parameters (Table 2), are of utmost importance. Fluid balance (including intake and output) should additionally be monitored. Skin integrity (including mucous membranes) and drug treatments (mainly diuretics) may impact the water and electrolyte balance, as well as diseases or conditions affecting renal perfusion (e.g., heart failure, chronic kidney disease) [33,67,68]. The evidence suggests that multicomponent interventions (including increased staff awareness, assistance with drinking, support using the toilet, and a greater variety of drinks on offer) may be effective [69]. Fluid therapy may be used for resuscitation, replacement, or maintenance of the fluid balance depending on the stage of illness. The National Institute for Health and Care Excellence (NICE) provides a set of interrelated algorithms for assessment, fluid resuscitation, routine intravenous maintenance, and replacement and redistribution of fluid and electrolytes [70]. Resuscitation may be needed in the case of considerable blood loss (e.g., surgery, injury) or plasma loss (e.g., burns, acute pancreatitis). Fluids are then immediately needed to maintain circulation and organ vital function [70]. Fluid maintenance may be initiated once the vital signs and urine output are normalized.

The maintenance of fluid and electrolyte balance is the key to prevent and treat dehydration. Maintenance aims to restore insensible losses and provide sufficient water and electrolytes to maintain the normal status of body fluid compartments and sufficient water to enable the kidneys to excrete waste products. Maintenance fluid prescriptions should target the daily requirements and replace ongoing abnormal losses. Requirements of patients receiving artificial nutrition are mostly covered though the artificial nutrition itself. Special attention is needed regarding overhydration, particularly in patients suffering congestive heart failure. The best route/method/fluid for administration depends on the severity/nature/acuteness of dehydration. Oral administration should be the preferred option, although parenteral administration of fluids may be mandatory in some cases. Such prescriptions must be changed to oral administration as soon as possible, as fluids prescriptions are often maintained too long, causing infects and oedema, especially in the postoperative phase. Subcutaneous infusions should be considered to manage chronic problems, particularly in the elderly [5]. A calculation of fluids requirements has been proposed by Chidester et al. [71], suggesting the prescription of 100 mL per kilogram body weight for first 10 kg, 50 mL per kilogram for next 10 kg, and 15 mL for the remaining kilograms of body weight. When body temperature is higher than 37 °C, 100–150 mL should additionally be given.

### 7.2. Oral/Enteral Replacement

Fluid and electrolyte requirements should be met via oral or enteral routes as far as possible when fluid resuscitation is not needed [70]. The first measure to replace fluid loss should be offering thickened liquids or food with high fluids content, whereas sodium-containing food and liquids must be avoided. As previously mentioned, the evidence level for such interventions is, however, weak [72]. Enteral liquid administration (via feeding tube) may be appropriate in the case of severe dysphagia or if maintaining fluid balance is not possible. Moderate dehydration (loss of 1.5–2% body weight) may easily be corrected with the administration of oral or enteral fluids, but already alters moods and causes decreased physical and cognitive performance [62,63,73,74]. Treatment for low-intake dehydration involves administration of hypotonic fluids [36], which will help correct the fluid deficit while diluting the raised osmolality. Oral rehydration therapy (aiming the replacement of electrolytes lost in volume depletion by diarrhoea or vomiting) and sports drinks are not indicated. Hydration status should be reassessed regularly until corrected, and then regularly monitored.

### 7.3. Parenteral Replacement

**Figure 7:**
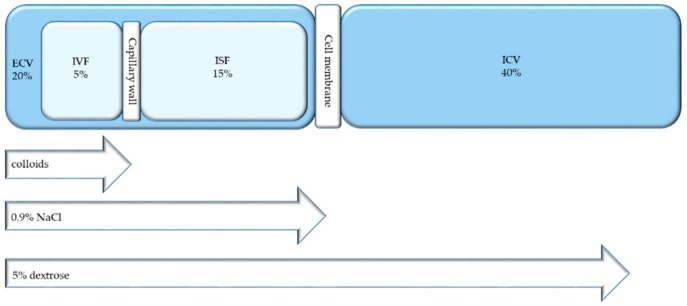
Infused fluids across the body compartments. ECV: Extracellular volume, ICV: Intracellular volume, IVF: Intravascular fluids, ISF: Interstitial fluids, NaCl: Sodium chloride.

The parenteral administration of (low sodium-containing) fluids is the method of choice in the case of severe dehydration (high amounts of fluid needed) or when intravenous access is anyway given for administration of medication or nutrition [36,70]. Intravenous hydration must, however, always be regarded to as a medical treatment and not as basic care. The risk and benefit balance must be cautiously considered. Figure 7 illustrates the distribution of different fluids within body compartments after infusion. Deficits in free water must be substituted quickly if hypernatremia lasts for shorter than 48 hours. To avoid oedemas, caused by fluids shifts, high-rate administration of intravenous hypotonic solutions must be prevented. As its cells accumulate osmolytes to maintain normal cell volume, the brain is especially endangered. Brain oedema may develop as a result of hasty administration of hypotonic solutions. Fluids must hence be administered progressively, over 12 to 48 h. Hyponatraemia lasting for more than 48 h have to be corrected slowly. Not more than half of the replacement should occur in the first 24 hours of treatment, during which one should look out extremely cautiously for brain oedema, the symptoms of which may be headache or seizures. The residual replacement may occur within the next 48 to 72 h. In the case of hypernatremia, fluid replacement is considered adequate when the urine sodium concentration is over 25 mmol/L.

## 8. Hypodermoclysis

Evidence has shown that adequate volumes of subcutaneous infusions are an effective treatment for dehydration, showing comparable rates of adverse effects to parenteral administration [36,75]. Many clinical trials have shown the safety and effectiveness of fluid infusion into the subcutaneous space using a fine cannula, called hypodermoclysis (HDC), making it a possible alternative to parenteral administration. HDC is particularly useful for the rehydration of patients with mild to moderate dehydration, who are cannot keep their fluid balance via the oral route [76,77,78]. Preferred sites for HDC are, for example, the anterior chest wall, lateral abdominal wall, inner tight, and back above or below shoulder blade. Recommendations for fluid infusion rates differ; that is, using gravity, up to 1,500 mL may be infused over 24 h at one site (rate of 20–80 mL/h), while rates of 82–148 mL/h have been recommended as well [32,79,80,81]. The authors’ clinical experience indicates the safety of infusion of 500 mL per hour. A study using radioactive labelled injection showed that absorption into the blood occurs within one hour [82]. Infusion solutions are chosen regarding the clinical condition of the patient (balanced solutions, saline solutions, dextrose solutions, or their combination). A study among dement nursing home residents suffering dehydration showed more agitation (80%) in subjects receiving parenteral fluids compared with those receiving subcutaneous fluids (37%), regardless of the volume of administered fluid or the improvement of dehydration [83].

Subcutaneous infusions with hyaluronidase (useful enzyme to break down hyaluronic acid, thus opening the interstitial space) usually permit a more rapid infusion rate, lower rate of moderate oedema, and more comfort for the patient. Even though there are no systematic studies available, administering up to 20 mmol/L potassium chloride appears unproblematic. Local adverse effects (e.g., oedema, erythema, and cellulitis) and systemic adverse effects (e.g., heart failure or hyponatremia) occur rarely and at similar rates compared with parenteral administration [78]. The effectiveness of both methods appears to be the same. The expense for HDC is about four times less than intravenous administration and demands reduced nursing care [83,84]. HDC use may be easily used in a community setting (nursing home or home), by patients and/or their caregivers [77]. HDC is suitable to prevent dehydration or to treat mild to moderate dehydration. Intravenous fluid replacement remains mandatory, however, to treat hypovolemic shock or severe dehydration.

### Colloids and (Balanced) Crystalloids

The volume of distribution and the metabolization of the solute are the two main factors to determine the plasma expansion capacity of a solution. Colloids are principally distributed within the intravascular space, while dextrose solutions are first metabolized and then distributed within the whole body water. Their volume expansion ability is hence limited and temporary. Resuscitation should thus be undertaken using colloids. Intravascular volume expansion is mostly done using crystalloids. If appropriated, dextrose solutions (5% or combined with saline) are used to provide free water. The expansion capacity of isotonic crystalloids, distributed within the extracellular fluids, is 20% to 25%. The remaining crystalloid volume is kept within the interstitial space. This overload of interstitial fluid has to be eliminated after the acute phase of the disease is over. Even if the use of crystalloid solutions is effective, the price to pay remains quite high. Additionally, the excessive use of sodium-containing crystalloids may cause oedema and further impair the clinical outcome. Dextrose solutions may cause potential harmful effects as well (e.g., hyponatraemia <130 mM). Hypochloraemic acidosis may occur as a result of the administration of saline, as they contain much higher concentrations of chloride compared with plasma. This may seriously impair the kidney function (reduced perfusion and glomerular filtration rates), the gastrointestinal function (mucosal acidosis, ileus), or other cellular dysfunction including impairment of mitochondrial function. Recent studies have shown the superiority of balanced electrolyte solutions (Hartmann’s or Ringer’s) on saline solutions replacing water and salt deficits, except for cases of gastric juice losses, where chloride losses are high [5]. Isotonic dextrose solutions should be used to replace deficits in free water. Colloid solutions such as albumin or hydroxyethyl starch should be used to expand volume, as they induce less salt/water overloads and oedemas than crystalloid solutions (e.g., dextrose 5%, 0.9% NaCl, and balanced crystalloids). The combination of both in varying proportions may be used in the clinical practice, depending on the patient’s condition. Newly published trials show balanced crystalloids to be superior on saline solutions in critical and non-critical care [85,86]. These trials showed a lower rate of the composite outcome of death from any cause, new renal-replacement therapy, or persistent renal dysfunction with balanced crystalloids than with saline (number needed to treat 1/94).

## 9. Conclusions

Dehydration is a relevant clinical problem with severe consequences, particularly in patients suffering from dysphagia, whose fluids’ intake is very restricted by the use of thickeners. At least in one-third of inpatients, dehydration is misdiagnosed and thus underestimated as well as undertreated. It is very important for the medical and nursing staff dealing with patients suffering dysphagia to be aware of dehydration, and work closely with speech-language pathologists and dietitians to place a nutrition and hydration care plan tailored to the needs and condition of each patient. Volume depletion, treated with isotonic fluids, and dehydration treated with hypotonic fluids, must be clearly taken apart. Rehydration should be treated by the oral (or enteral) route whenever possible. Parenteral rehydration must be used in severe cases. The use of HDC may be considered in the case of mild dehydration when there are contraindications for oral/enteral treatment. It represents an effective and safe treatment option, especially in patients with dysphagia.

## Figures and Tables

**Table 1:** Electrolyte concentrations in the human body [5,52].

Electrolyte	Plasma (mmol/L)	Extracellular Volume (mmol/L)	Intracellular Volume (mmol/L)
Sodium	135–145	142–155	10–18
Potassium	3.5–5.3	4.0–5.5	120–145
Calcium	2.2–2.6	2.2–2.5	1.5
Chloride	95–105	98–108	2–6
Magnesium	0.8–1.2	0.7–1.2	15–25
Phosphate	0.81–1.45	0.7–1.3	8–20
Bicarbonate	22–30	22–30	10

**Table 2:** Single signs, symptoms, and laboratory tests to identify dehydration [33,58,59].

Assessment of Hydration Status	Feasibility of Test	Scientific Value
**Signs and symptoms**		
Seated systolic blood pressure ≤100 mmHg	H	H
Blood pressure change supine/standing ≥20 mmHg	H	H
Thirst sensation	H	M
Dark urine colour	H	M
**Laboratory tests**		
Urine specific gravity ≥1.025	H	H
Blood urea nitrogen/creatinine ratio ≥20	M	H
Blood osmolality calculated ≥300 mmol/kg	M	H
Haematocrit/haemoglobin ratio	M	M
Mean corpuscular volume	M	M
Serum sodium concentration >150 mmol/L	M	M
**Additional measurements** (mainly for scientific purposes)		
Total body water (isotope dilution)	L	M
Total body water (bioelectrical impedance analysis)	H	M
Fluid volumes and ionic content (neutron activation analysis)	L	M
Blood osmolality (measured)	M	H
Urine osmolality	H	H
Salivary osmolality	H	H
Tear osmolality	M	M
Intraocular pressure (measured)	M	L

H = high; M = medium; L = low.
